# Imine-Linked Covalent Organic Framework with a Naphthalene Moiety as a Sensitive Phosphate Ion Sensing

**DOI:** 10.1021/acsami.1c24555

**Published:** 2022-05-03

**Authors:** Mohaddeseh Afshari, Mohammad Dinari, Hossein Farrokhpour, Félix Zamora

**Affiliations:** † Department of Chemistry, Isfahan University of Technology, Isfahan 84156-83111, Islamic Republic of Iran; ‡ Departamento de Química Inorgánica, Facultad de Ciencias, Universidad Autónoma de Madrid, Campus de Cantoblanco, Madrid 28049, Spain

**Keywords:** covalent organic framework, ion sensing, phosphate ion, photoluminescence, DFT calculation

## Abstract

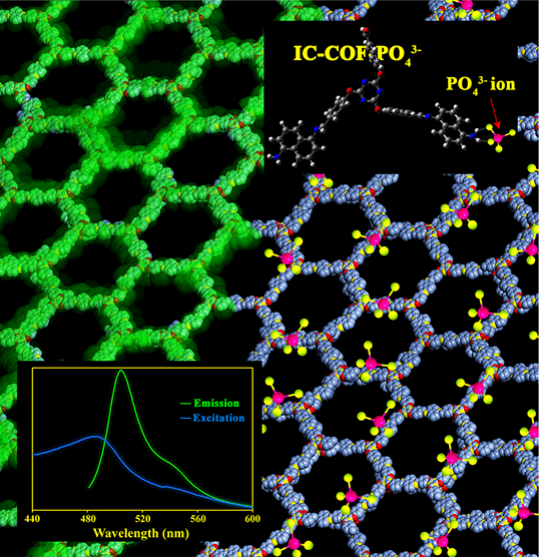

Due to the excellent ion-sensing potential of covalent organic frameworks (COFs), the new imine-linked conjugated COF (IC-COF) is synthesized through a water-based synthesis reaction between 1,5-diaminonaphthalene and 2,4,6-tris(4-formylphenoxy)-1,3,5-triazine to create a luminescence sensor. It is noteworthy that the green synthesized IC-COF shows excellent selectivity to phosphate ions (PO_4_^3–^) with a detection limit of 0.61 μM. The recyclability performance of IC-COF is high, indicating that it can be reused without a significant reduction in performance (5.2% decline after 5 cycles). Theoretical calculations using the density functional theory are performed on the IC-COF–PO_4_^3–^ and IC-COF–Cu^+^ complexes to explore the sensing mechanism. The fluorescence quenching in the presence of PO_4_^3–^ ions is attributed to the difference between PO_4_^3–^ binding sites to the IC-COF compared to Cu^+^, which leads to the considerable change in the IC-COF absorption spectrum from 400 to 600 nm.

## Introduction

Covalent organic frameworks (COFs) built by connecting molecular building units with dynamic covalent bonds represent an exciting new type of porous crystalline polymers.^1−5^ The targeted design^6^ and synthesis of COFs have attracted tremendous attention owing to their outstanding properties such as high physicochemical stability,^7−9^ a large specific surface area, and highly ordered pore structures.^10−14^ That is why COF materials have been utilized in many applications such as chemical carrier,^15^ fluorescence recognition,^16−18^ hydrogen evolution,^19,20^ sensors,^21^ heterogeneous catalysis,^22,23^ and proton conduction,^24^ to name a few. Some synthesized COFs have shown extraordinary luminescence properties and great potential for sensing applications.^25^ The exceptional feature of these structures amplifies the generated luminescence signal in their conjugated framework, leading to higher luminescence intensity.^26^ Also, a sequence of multiple functional groups in an extensive framework enables it to react with trace amounts of analytes.^27^ COF-based fluorescence sensors have unique properties such as high quantum efficiency, signal amplification effect, fast response time, and a long luminescent lifetime, which has led to significant interest among scientists in using these polymers as probes for various anions, cations, and small organic molecule detection.^28^

Phosphate (PO_4_^3–^) is one of the most effective anions in aquatic environments and biological systems due to its participation in most metabolic processes. As a mineral nutrient, PO_4_^3–^ ion promotes the growth of marine organisms. It is well known that an excessive amount of phosphate ions in water can intensify eutrophication and induce plankton and aquatic plant blooming, depleting the dissolved oxygen in the water and resulting in the deterioration and death of marine organisms.^29^ In addition to aqueous systems, phosphate plays a crucial role in biological processes such as bone mineralization, information transfer, storage and transfer of energy, and gene construction.^30,31^ Finally, it is worth mentioning that the appearance and progression of many diseases in the human body (e.g., hypophosphatemia, osteoporosis, hyperparathyroidism, Fanconi syndrome, and vitamin D deficiency) can be due to the presence/absence of the right concentration of phosphate ions.^32^ Therefore, determining the concentration of phosphate ions is very important from biological and environmental aspects. There are many available techniques for detecting phosphate.^33^ Unlike traditional analysis methods, fluorescence sensing of phosphate ions, especially with new probes of porous polymers, is a decent method due to its distinctive features, including fast response, portability, and easy operation.^30,31,34^

The main problem with using this technique is the need to use probes with complicated, expensive, and time-consuming synthesis methods in the presence of toxic organic solvents.^35^ Given the above, the development of probes with high sensitivity and selectivity, using green synthesis methods without the intervention of hazardous organic solvents, to detect the vital ions remains a challenge to this day.

**Figure 1:**
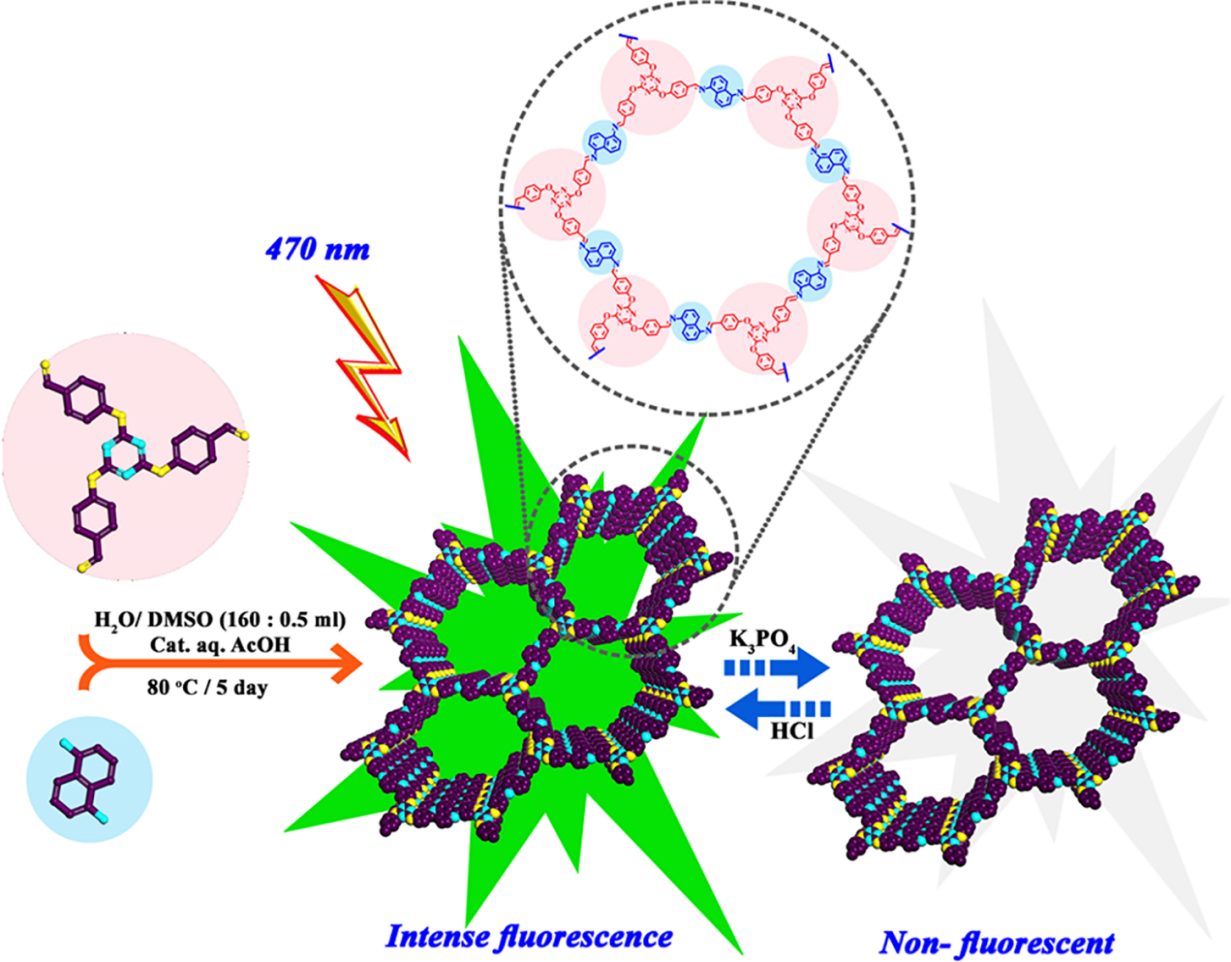
Synthesis of IC-COF and the effect of phosphate ions on its fluorescence emission.

Herein, the reusable fluorescence sensor of imine-linked conjugated COF (IC-COF), as a novel PO_4_^3–^ ion receptor, was designed and synthesized (Figure 1). The IC-COF was prepared through a solvothermal condensation reaction between 1,5-diaminonaphthalene and 2,4,6-tris(4-formylphenoxy)-1,3,5-triazine (TFPT) in the presence of green solvents. The fluorescence of IC-COF switched to turn-off upon the addition of phosphate or carbonate and had negligible or no response to other anions and cations.

Unlike conventional preparation methods of imine-based COF, which performed under approximately harsh conditions, IC-COF was synthesized using a green synthesis method with high efficiency through a Schiff-base condensation reaction of synthesized TFPT with 1,5-diaminonaphthalene. In a general preparation, the reaction was performed in a water solvent and DMSO as a cosolvent, with a volume ratio of 160 to 0.5 in the presence of aqueous acetic acid at 80 °C. The experimental preparation parameters were adjusted to enhance COF crystallinity.

**Figure 2:**
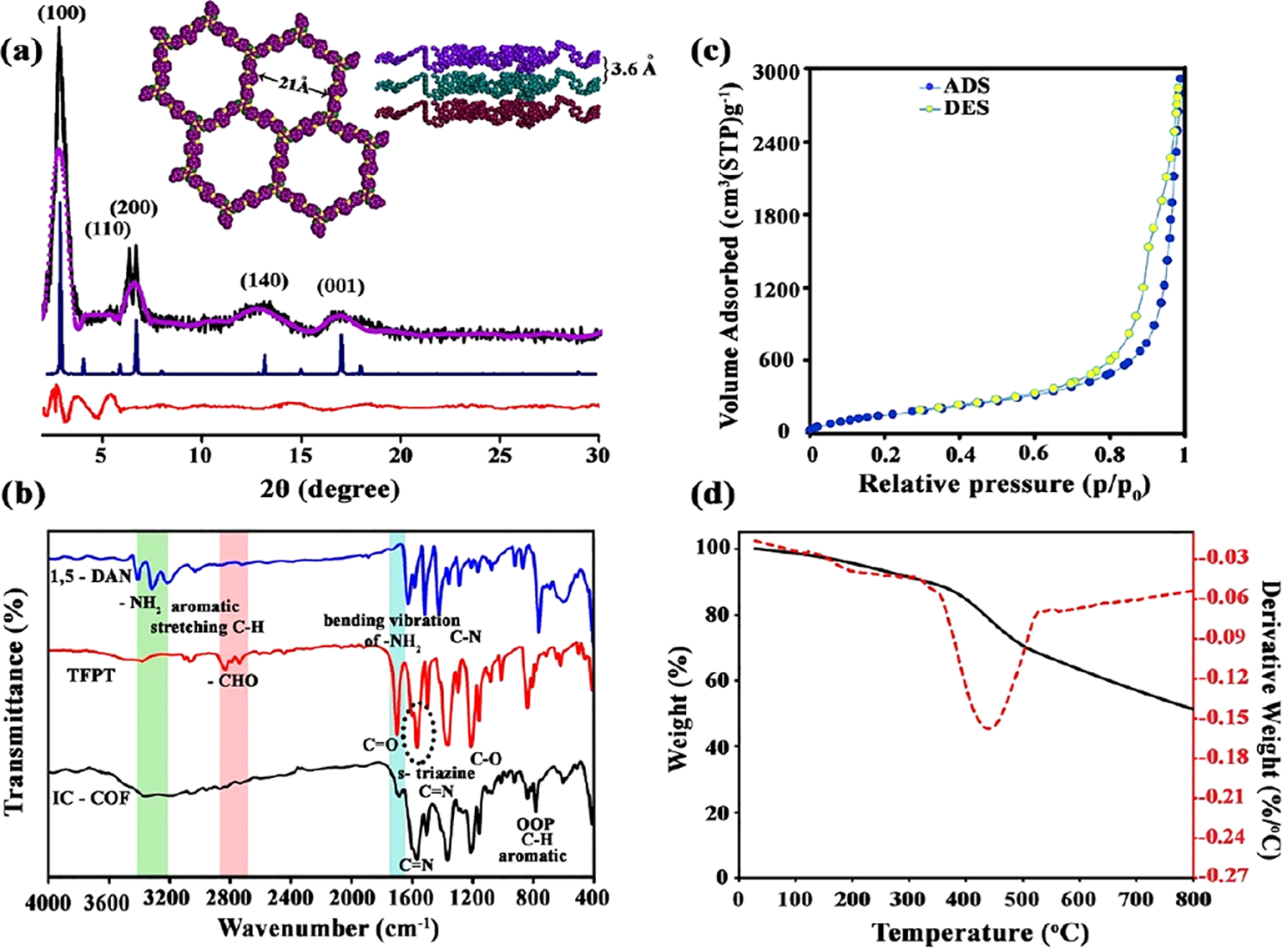
(a) Experimental (black), Pawley refined (purple), and simulated eclipsed AA stacking (blue) PXRD patterns of IC-COF. The difference plot between the experimental and refined patterns is shown in red. Inset: the eclipsed stacking structure proposed for IC-COF. C, purple; O, green; N, yellow; (b) FTIR spectra of IC-COF and its constituent monomers; (c) nitrogen adsorption and desorption isotherm curves (77 K) of IC-COF; (d) TGA and DTG curves of IC-COF under argon atmosphere.

PXRD analysis was carried out to determine the IC-COF structure. Figure 2a shows a strong diffraction peak at 2.86° and a set of additional weaker peaks centered at 6.40, 6.66, 13.37, and 17.01°, assigned to 100, 110, 200, 140, and 001 facets, respectively. To clarify the crystalline structure of synthesized IC-COF, 2D layered structures with staggered AB (Figure S1a,b) and eclipsed AA (Figure S2a,b) stacking were modeled using version 7.0 of the Materials Studio software.^36^ The experimental XRD pattern was better matched with the simulated pattern obtained from the AA eclipsed structure (space group *P*6/*m*) rather than AB staggered ones. Accordingly, the simulated eclipsed model was used for the Pawley refinement. The optimal lattice parameters obtained by refinement results for a hexagonal unit cell are presented as follows: *a* = *b* = 30.37 Å, *c* = 4.19 Å, α = β = 90°, and γ = 120°.

The comparison of the FTIR spectra of 1,5-diaminonaphthalene, TFPT, and IC-COF (Figure 2b) confirms the formation of the polyimine structure in IC-COF. Thus, the peaks at 2830 and 2740 cm^–1^ can be attributed to the CHO stretching vibration, and the sharp peak appearing at 1701 cm^–1^ is related to the C=O stretching vibration of the aldehyde groups. The bands located at 1563 and 1494 cm^–1^, in the TFPT spectrum, correspond to the quadrant and semicircle stretching of the *s*-triazine rings.^37^ The peak corresponding to the primary amine of 1,5-DAN also appeared at 3409 and 3315 cm^–1^. The disappearance of the absorption bands belonging to the carbonyl and C–H stretching vibration of aldehyde groups and −NH stretching vibration of amine groups in the final structure indicates the completion of the Schiff-base reaction and the formation of IC-COF. Besides, the peak at 1598 cm^–1^ can be assigned to the C=N stretching vibration, which are characteristic skeleton peaks of IC-COF. The peaks at 1363 and 1297 cm^–1^ can be assigned to aromatic C–N and C–C ring stretching vibration, respectively. The peak at 780 cm^–1^ corresponds to the out-of-plane bending vibration of aromatic C–H.

Nitrogen adsorption/desorption experiments determined the porosity of IC-COF. The specific surface area of IC-COF from the Brunauer–Emmett–Teller model was 647 m^2^ g^–1^ (Figure 2c). Based on a single-point measurement (at *P*/*P*_0_ = 0.99), the total pore volume was determined to be 4.51 cm^3^ g^–1^. The resulting relatively low surface area may be due to some amount of unreacted monomers or solvent molecules at the cavities.^27^ The first weight-loss stage observed in the thermogravimetry/derivative thermogravimetry (TG/DTG) (TG/DTG) curves can confirm this issue.

The TGA and DTG curves of IC-COF under argon atmosphere are shown in Figure 2d, and related data are listed in Table S1. According to the observations, the as-prepared COF shows good thermal stability. The observations exhibit two main weight-loss steps in the TGA curve. The first weight loss begins with a constant gentle slope higher than 100 °C and continues to 400 °C (about 14%). The second step occurs from 400 °C and continues up to 800 °C. The imine connections were broken at this stage, and the covalent framework was decomposed into the primary monomers.

**Figure 3:**
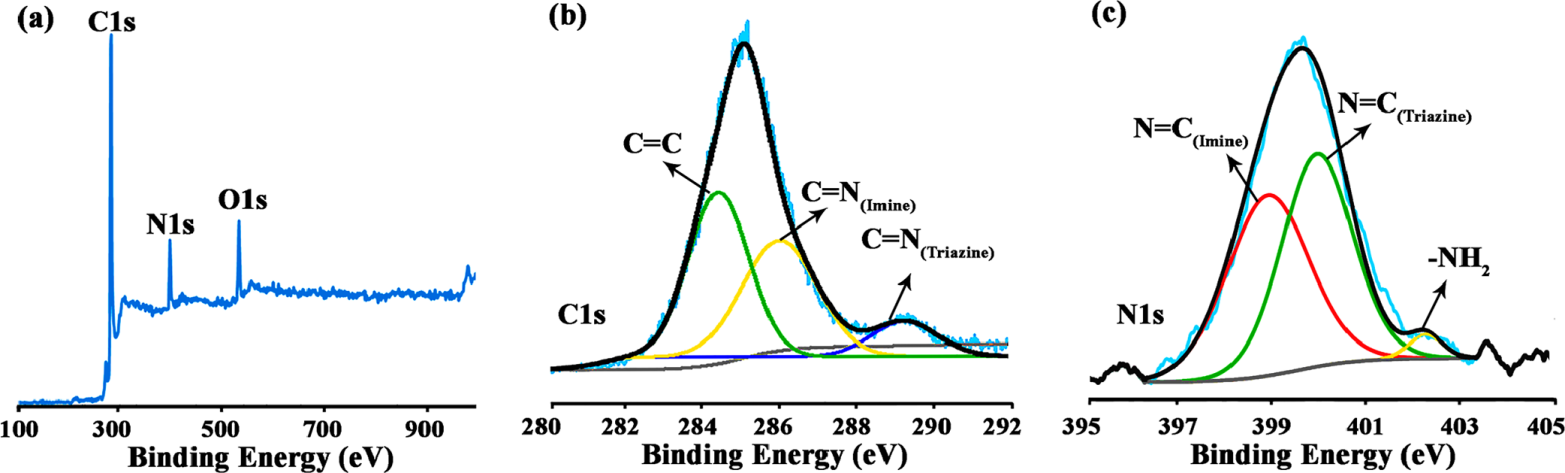
XPS survey spectrum (a) and the high-resolution spectra of C 1s (b) and N 1s (c) for IC-COF.

Furthermore, the formation of imine linkage in IC-COF was demonstrated by X-ray photoelectron spectroscopy (XPS). The peaks of C 1s, N 1s, and O 1s appeared in the broad scan XPS spectrum (Figure 3a). In high-resolution C 1s XPS spectra, the three peaks with binding energy at 284.3, 286.3, and 289.6 eV were assigned to C=C and C=N in the imine bond and the triazine ring, respectively (Figure 3b). The N 1s band was deconvoluted into three peaks (Figure 3c), at 398.8, 400.0, and 402.2 eV. The peaks at 398.8 and 400.0 eV were assigned to the nitrogen atoms in the imine bond triazine ring and the dangling protonated −NH_2_ bond, respectively, while the peak at 402.2 eV was related to the unreacted terminal amino groups at the end of the COF framework segment. These results confirm the successful synthesis of IC-COF.

**Figure 4:**
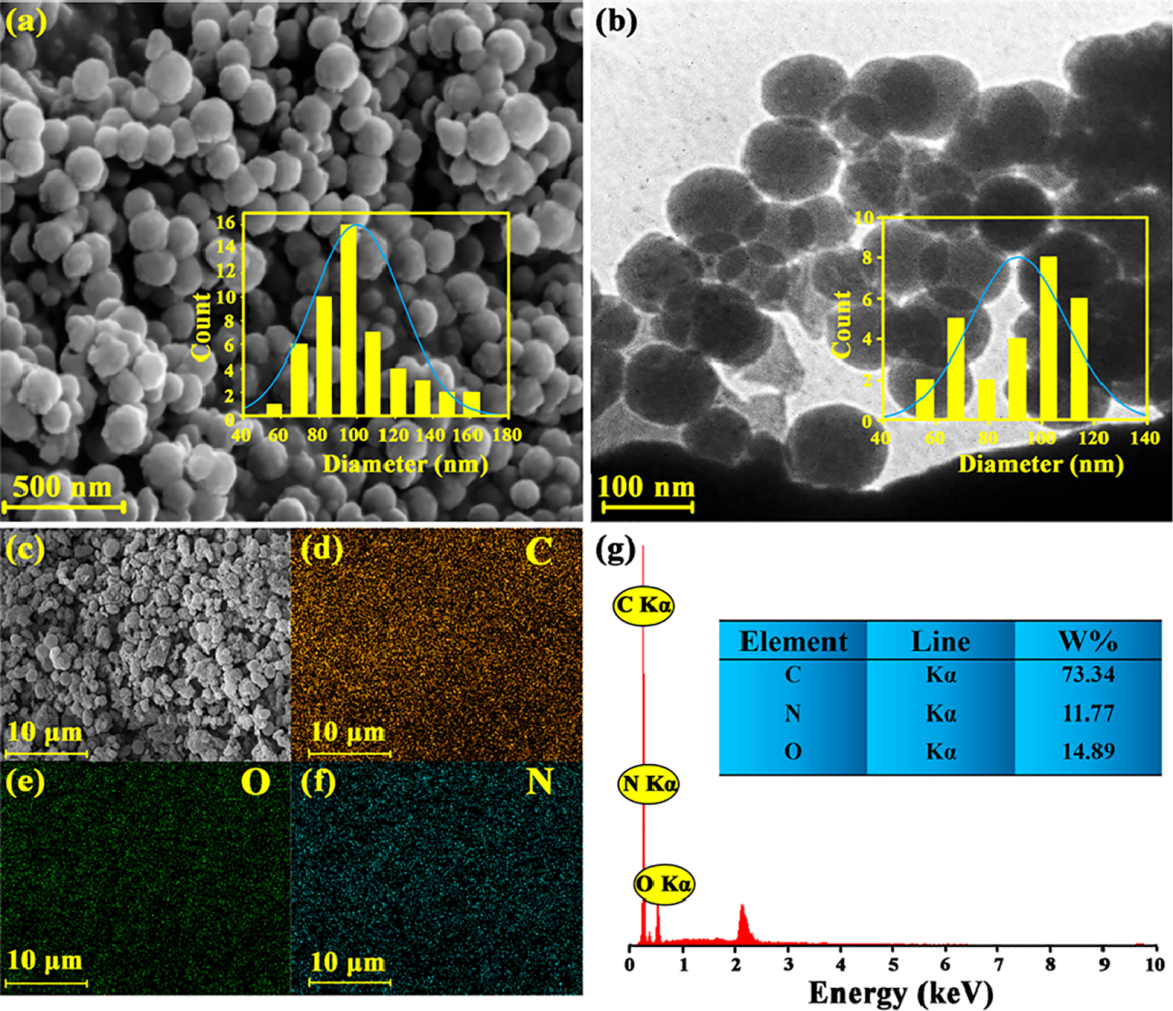
(a) FESEM image; (b) TEM image. Inset: their histogram of IC-COF particle size distribution; (c–f) corresponding EDS elemental mappings of C, N, and O; (g) EDS pattern of synthesized IC-COF.

Field emission scanning electron microscopy (FESEM) images of IC-COF (Figures 4a and S3a) confirm its nanosphere morphology with diameters of ca. 98 ± 26 nm and a good homogeneous distribution. The formation of spherical morphologies during the reaction process may be rationalized to the hydrophobicity of the synthesized framework and the inward orientation of the sheets. In comparing the various examined methods for synthesizing IC-COF (Figure S4) with the introduced green method (as the optimal method), it was observed that the use of the aqueous phase as a solvent could be a way to acquire a regular and uniform structure through intramolecular rotation.

On the other hand, the self-assembly process in the reversible polymerization of COFs was affected by lower surface free energy and the π–π stacking interactions between adjacent layers, leading to spherical structures.

The transmission electron microscopy (TEM) images of synthesized IC-COF also display the spherical morphology with an average diameter of 91 ± 17 nm (Figures 4b and S3b). Figure 4c–f shows the energy-dispersive X-ray spectroscopy (EDX) elemental mapping for IC-COF. It observes a homogeneous uniform distribution of elements throughout the framework. There is also a good correlation between the intensity of the observed peaks in EDX analysis and the obtained values from elemental analysis (Figure 4g).

The UV–vis absorption spectra of dispersed IC-COF in common organic solvents, including DMSO, DMF, ethanol, THF, and cyclohexane, are shown in Figure S5. The optimal excitation wavelength in each solvent was evaluated by the fluorescence spectrum of IC-COF in the range of ±30 nm of the obtained maximum absorption wavelength from UV–vis spectra. The fluorescence spectra recorded at the optimum excitation wavelength for each solvent are shown in Figure S6, and the relevant data are listed in Table S2. The results presented that the fluorescence emission intensity of IC-COF dispersed in different solvents had a remarkable difference. Dispersed IC-COF in DMSO showed the highest emission intensity at 505 nm, upon the excitation at 470 nm, among the examined solvents (Figure S6). The emission intensities of IC-COF in different solvents decreased in the order of DMSO, DMF, THF, ethanol, and cyclohexane. The weakest emission intensity was observed in the case of cyclohexane, which we think is due to differences in the polarity of solvents. Thus, the subsequent experiments were conducted in the DMSO medium. The photographic images of the test tubes containing IC-COF in different solvents, before and after irradiation at 470 nm, are shown in Figure S7.

In contrast, 1,5-diaminonaphthalene and TFPT do not show fluorescence emission upon excitation at 470 nm (Figure S8). These significant differences in the fluorescence properties between IC-COF and the corresponding monomers are likely due to the extensive conjugated structure in the IC-COF framework and confirm the properties of this conjugated framework to be used as a fluorescence sensor.^36^

**Figure 5:**
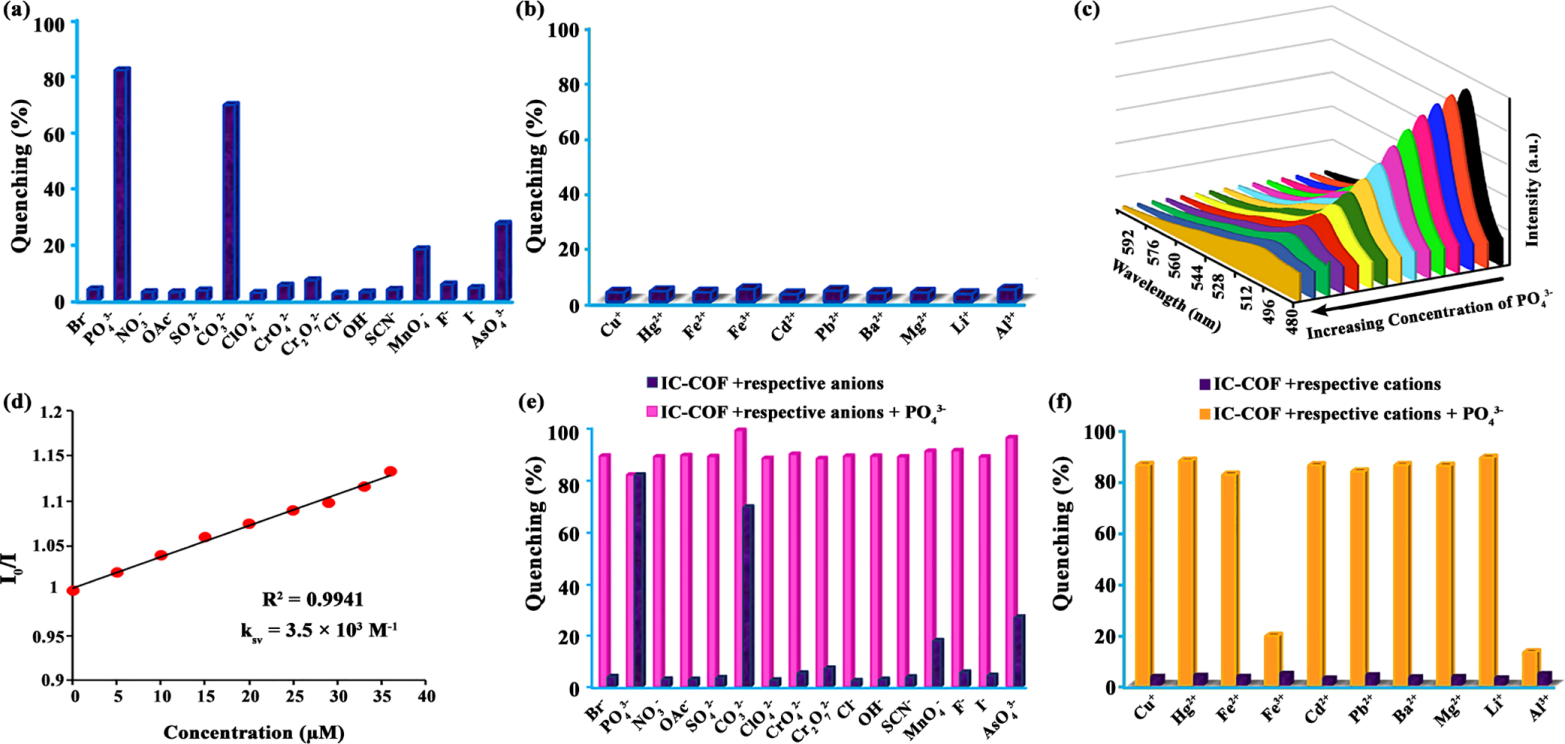
Percentage fluorescence quenching of IC-COF upon the addition of multiple anions (a) and cations (b); fluorescence spectra of the IC-COF suspensions upon the gradual treatment with PO_4_^3–^ (0–37 μM), excited at 470 nm (c); linear correlation for the plot of *I*_0_/*I* as a function of PO_4_^3–^ concentration (d); percentage fluorescence quenching of IC-COF by the PO_4_^3–^ ions in the presence of other anions (e) and cations (f).

The highly fluorescent nature of IC-COF and its many active sites in its structure prompted us to test the IC-COF ability for fluorescence sensing. The fluorescence quenching efficiencies of IC-COF for various ions are shown in Figure 5. The luminescence intensity of IC-COF at the emission wavelength of 505 nm remained unchanged or negligibly quenched after adding multiple anions and cations. In contrast, significant fluorescence quenching was observed for PO_4_^3–^, CO_3_^2–^, and AsO_4_^3–^ ions. Interestingly, the fluorescence intensity of IC-COF was reduced by 82% in the presence of only 10 μL of 0.01 M phosphate solution.

The concentration gradient experiments were performed by changing the concentration of PO_4_^3–^ and CO_3_^2–^ to further evaluate the sensor’s sensitivity as one of the most critical parameters for a fluorescence sensor. It was observed that with the increase in the concentration of PO_4_^3–^ or CO_3_^2–^, the fluorescence intensity of IC-COF gradually decreased (Figures 5c and S9a). The alteration in relative fluorescence intensity as a function of PO_4_^3–^ concentration is indicated in Figure 5d (Figure S9b for CO_3_^2–^ anion). The *k*_SV_ values obtained from the Stern–Volmer equation for PO_4_^3–^ and CO_3_^2–^ were 3.5 × 10^3^ and 3.1 × 10^3^ M^–1^, respectively. The detection limits for PO_4_^3–^ and CO_3_^2–^ anions were calculated as 0.61 × 10^–6^ and 1.2 × 10^–6^ M based on 3σ/*k*, respectively, which are acceptable compared to those reported for detecting these anions^38,39^ (Table S3), in which σ is the standard deviation of three repeated luminescence measurements of the blank, and *k*_SV_ is the slope of the linear equation. The PO_4_^3–^ ions are the most effective quencher anion with a better detection limit, indicating a very high IC-COF sensitivity toward this anion. Because of the greater sensitivity of IC-COF to PO_4_^3–^ ions, other tests were performed with this anion.

Additional experiments in the presence of other interfering ions were carried out to evaluate the applicability of IC-COF for phosphate detection. The results showed that in the presence of different anions, some factors such as dilution of the solution slightly changed the intensity of luminescence and did not significantly interfere with the fluorescence quenching process of IC-COF by PO_4_^3–^ ions (Figure 5e). Similar results were observed among cationic interfering species, except in Al^3+^ and Fe^3+^. This phenomenon could be due to the higher tendency of these ions to form the complex with the PO_4_^3–^ ions and lack of access to free phosphate ions (Figure 5f).

To study the quenching effect of phosphate ions at various pHs, the phosphate aqueous solution in pHs 3 to 10 was prepared using Tris buffer as a standard buffer. For this purpose, 5 μL of 0.01 M phosphate solution (with different pHs) was added to 2 mL of the IC-COF mother solution and its fluorescence emission was recorded. The results showed no significant shift in the fluorescence spectrum in the pH range of 3–7. A slight increase in the quenching effect may also be due to the increase in the relaxation time, which reduces the emission rate, and, consequently, the intensity of emission decreases. However, at pH 8–10, more emission quenching was observed with a red-shift. At alkaline pHs, the higher tris(hydroxymethyl)aminomethane concentration leads to the interaction between the amine group of Tris buffer and the end functional groups of IC-COF that could displace the absorption bands. The effect of pH on the quenching of IC-COF fluorescence property by phosphate ion is depicted in Figure S10.

**Figure 6:**
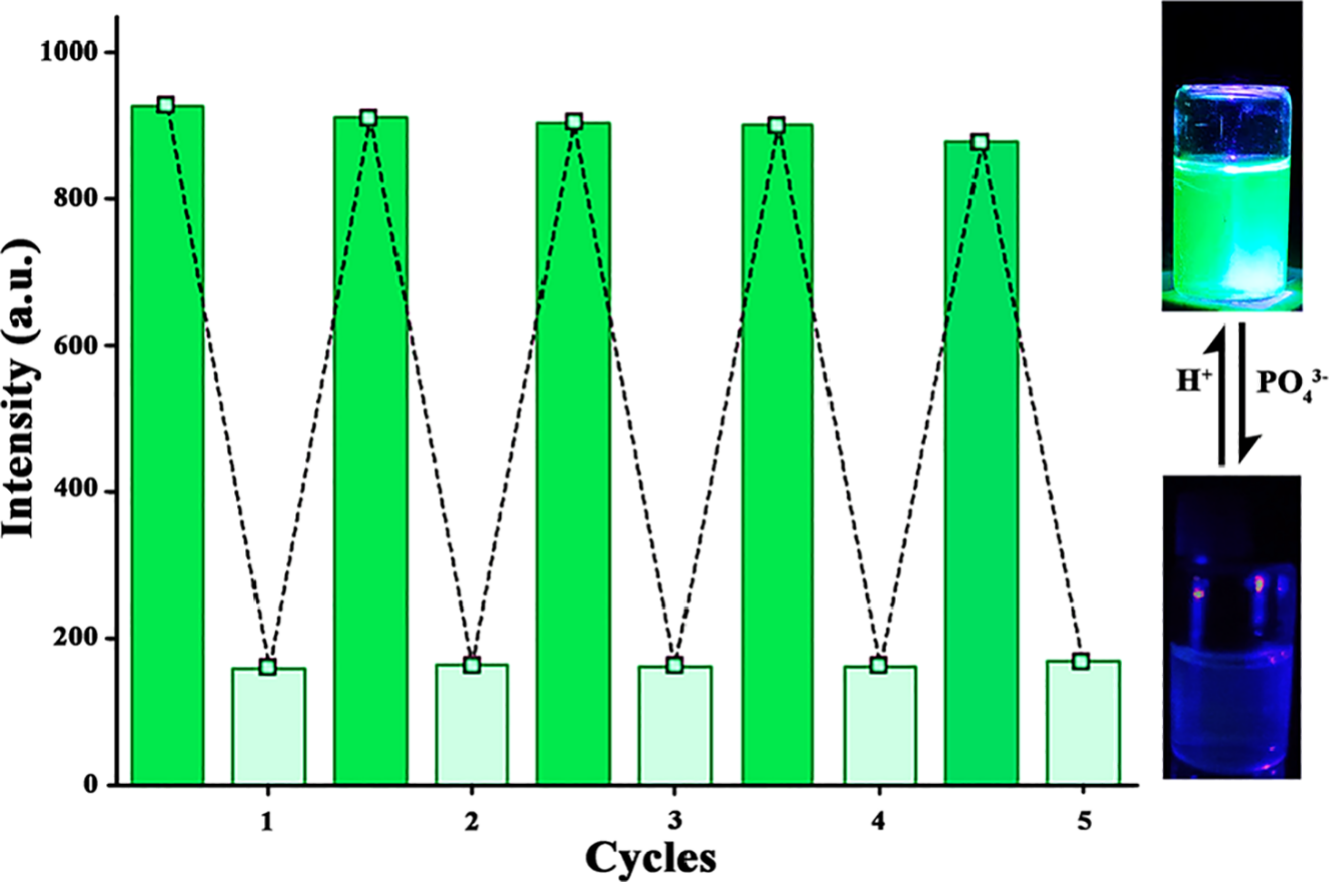
Recovery cycles of the phosphate ion are quenched by COF-IC sensor upon the addition of HCl.

Reusability is one of the most influential parameters in evaluating the capability of a fluorescence sensor. The observations showed that IC-COF quickly recovered its fluorescence emission by adding 3 μL of 0.1 M HCl aqueous solution (Figure 6), providing the possibility of selective and repeatable sensing of PO_4_^3–^ ions in the application. The significant IC-COF fluorescence quenching in the presence of the PO_4_^3–^ ions dramatically contrasts with the observed behavior versus metal ions such as Cu^+^, as a singlet electron cation.

Density functional theory (DFT) calculations were used to investigate the experimental observations about the IC-COF interaction with PO_4_^3–^ and Cu^+^ ions. These results can explore two pivotal points that are not accessible from experimental observations: (i) the determination of the best site of the selected IC-COF for the interaction with PO_4_^3–^ and Cu^+^ and (ii) the effect of the interacting ions on the IC-COF absorption spectrum. The DFT functional details of basis sets and the explanations about the preferred interaction sites of the selected IC-COF are provided in the Supporting Information (Figures S11 and S12).

**Figure 7:**
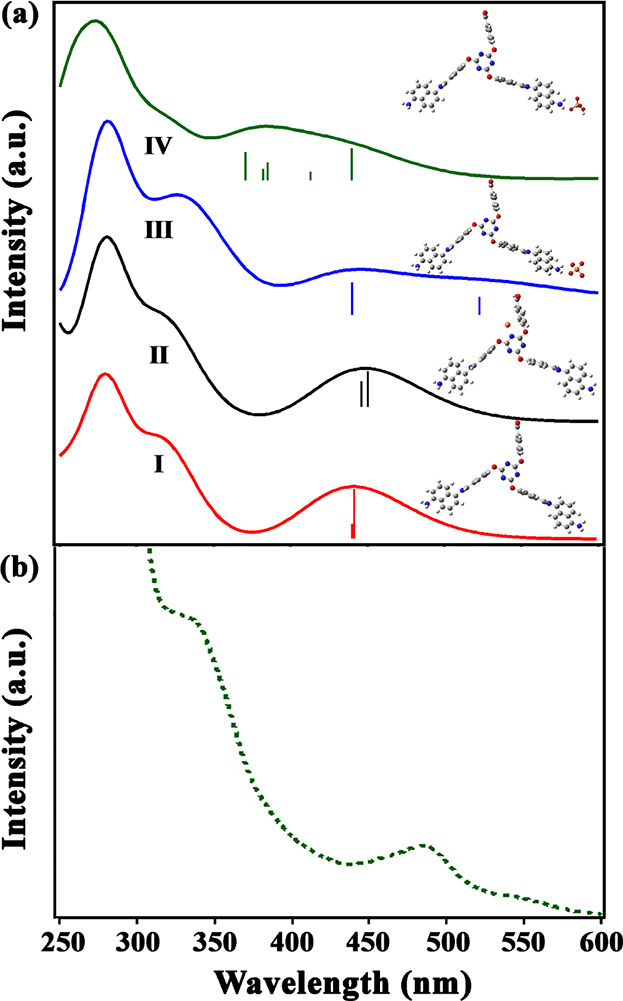
(a) Calculated absorption spectra of the (I) IC-COF, (II) IC-COF–Cu^+^, (III) IC-COF–PO_4_^3–^, and (IV) [(IC-COF–H)^−^/HPO_4_^2–^] in the DMSO solvent. (b) Part of the experimental spectrum of the IC-COF in the DMSO solvent.

Figure 7 shows the calculated absorption spectra of the bare IC-COF, IC-COF–PO_4_^3–^, and IC-COF–Cu^+^ in the DMSO solvent and compares them with the experimental spectrum of IC-COF in the range of 250–600 nm (the experimental UV–vis spectra of IC-COF–Cu^+^ and IC-COF–PO_4_^3–^ complexes are also shown in Figure S13). Based on the experimental observations, the intensity of the peak appearing at 400–500 nm was significantly reduced in the presence of PO_4_^3–^ ions. At the same time, no change in the IC-COF absorption spectrum was observed in the presence of Cu^+^ ions. These results were confirmed with calculated absorption spectra of IC-COF–PO_4_^3–^, IC-COF–Cu^+^, and IC-COF. The important absorption lines of the bare IC-COF and its complexes are shown in Figure 7a in the range of 450–550 nm.

**Figure 8:**
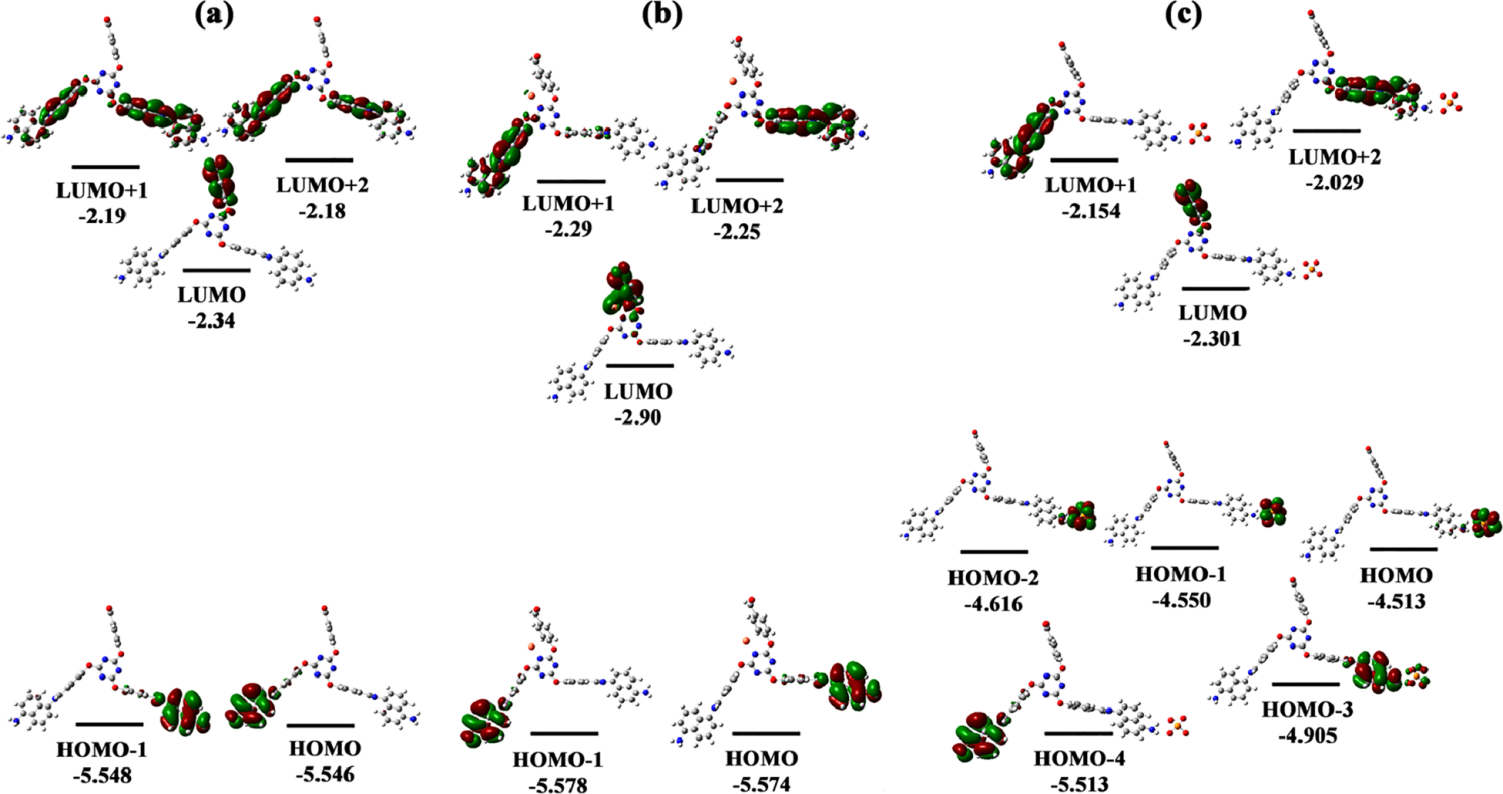
Energy diagram of some of the molecular orbitals of (a) IC-COF, (b) IC-COF–Cu^+^, and (c) IC-COF–PO_4_^3–^ involved in the main electronic configuration of their absorption lines is shown in (a). The numbers are the orbital energies in eV.

Based on the additional details explained in the Supporting Information, the observed excitation in the range of 400–500 nm is associated with the electron excitation from the diaminonaphthalene to the phenyl moiety of the TFPT structure. The bare IC-COF absorption spectrum observed two absorption lines at 439.7 and 441.2 nm. These electron excitations are related to the HOMO → LUMO + 1, HOMO → LUMO + 2, HOMO – 1 → LUMO + 1, and HOMO – 1 → LUMO + 2 excitations (Figure 8a).

The IC-COF emission is related to the transition from these excited states to the ground state of the molecule because of the emitted light wavelength (505 nm). Two absorption lines (450.1 and 445.9 nm) in the IC-COF–Cu^+^ complex are mostly related to the HOMO – 1 → LUMO + 1 and HOMO → LUMO + 2 excitations, respectively (Figure 8b). When PO_4_^3–^ replaces Cu^+^, a considerable change in the absorption spectrum is observed owing to the change in the interaction site of PO_4_^3–^ compared to Cu^+^. In the IC-COF–PO_4_^3–^ complex, one absorption line shifts to a higher wavelength (522.0 nm), decreasing the peak intensity. The IC-COF–PO_4_^3–^ absorption lines (439.7 and 522.0 nm) are related to HOMO – 4 → LUMO + 1 and HOMO – 3 → LUMO + 2, respectively (Figure 8c). The increase in the number of excited states in the IC-COF–PO_4_^3–^ absorption lines compared to the IC-COF–Cu^+^ complex is due to three PO_4_^3–^ orbitals among the IC-COF molecular orbitals (Figure 8). One of the absorption lines that shifts to the higher wavelengths in the IC-COF–PO_4_^3–^ complex reduces the number of excited molecules, which is the main reason for the decrease in the emission intensity of the IC-COF–PO_4_^3–^ complex compared to the bare IC-COF and IC-COF–Cu^+^ complex.

The other possibility between PO_4_^3–^ and IC-COF is the proton transfer from the terminal NH_2_ groups of the IC-COF to PO_4_^3–^ and the formation of the imine bond. The calculated absorption spectrum of the [(IC-COF–H)^−^/HPO_4_^2–^] complex has been shown in Figure 7a. As shown in the figure, the proton transfer shifts the 441 nm absorption line to the lower wavelength and the intensity of [(IC-COF–H)^−^/HPO_4_^2–^] absorption spectrum decreases from 400 to 500 nm. Therefore, the proton transfer mechanism can also be considered as the other alternative reason for the quenching of IC-COF emission in the presence of PO_4_^3–^. The 439.62 nm absorption line in the [(IC-COF–H)^−^/HPO_4_^2–^] spectrum is due to the HOMO – 2 → LUMO + 1 excitation. The energy diagram of [(IC-COF–H)^−^/HPO_4_^2–^] has been shown in Figure S14.

## Conclusions

In summary, this work presents a simple and efficient approach to synthesizing imine-based IC-COF using green solvents. The presence of the naphthalene moiety in the planar structure of IC-COF seems to play a fundamental role in its luminescence properties. Thus, IC-COF shows an intensive and phosphate ion quenchable luminescent emission, with a *k*_SV_ of 3.5 × 10^3^ M^–1^. On the other hand, other anions did not show a significant interference in the quenching efficiency of IC-COF by phosphate ions. Additionally, IC-COF can easily recover by adding dilute hydrochloric acid, which leads to frequent applicability. Theoretical calculations show that the absorption spectra of IC-COF versus IC-COF–Cu^+^ and IC-COF–PO_4_^3–^ display a shift in one of the absorption lines to the higher wavelength and a decrease in intensity. Therefore, this is the main reason for the quenching observed for IC-COF–PO_4_^3–^ compared to IC-COF and IC-COF–Cu^+^. This behavior can be attributed to the considerable decrease in excited molecules.

## Supporting Information Available

Additional details on synthetic procedures, materials, and methods; characterization data; anion sensing procedure; details of theoretical calculations; unit cell and eclipsed and staggered crystal lattice packing of IC-COF; FE-SEM and TEM images of IC-COF, FE-SEM images of the obtained product from another methodes; UV-Vis spectra, and fluorescence spectra of IC-COF suspension in various solvents; digital images of IC-COF suspension in various solvents; comparison between fluorescence spectra of IC-COF and its constituent monomers in DMSO; fluorescence spectra of the IC-COF suspensions upon the gradual treatment with CO32- and the linear curve of I0/I vs. CO32- concentration ; effect of pH on the quenching of IC-COF fluorescence emission; TGA and DTG specific values of IC-COF; excitation and emission parameters of the IC-COF; and comparison of LOD of various probes (PDF)
