# Supplementary material for: Imine-Linked Covalent Organic Framework with a Naphthalene Moiety as a Sensitive Phosphate Ion Sensing
Source: ACS Appl Mater Interfaces. 2022 May 3;14(19):22398–406. doi: 10.1021/acsami.1c24555 (PMC9121346; doi:10.1021/acsami.1c24555)
Supplement: Supplementary file 1 — am1c24555_si_001.pdf [file am1c24555_si_001.pdf]

Supporting Information

An Imine-Linked Covalent Organic Framework with a Naphthalene Moiety as a Sensitive Phosphate Ion Sensing

Mohaddeseh Afshari,[a] Mohammad Dinari, *[a] Hossein Farrokhpour, *[a] Félix Zamora *[b]

[a] M. Afshari, Dr. M. Dinari, Prof. H. Farrokhpour
Department of Chemistry
Isfahan University of Technology
Isfahan, 84156-83111, Islamic Republic of Iran
E-mail: dinari@iut.ac.ir, h-farrokh@iut.ac.ir

[b] Prof. F. Zamora
Departamento de Química Inorgánica, Facultad de Ciencias
Universidad Autónoma de Madrid
Campus de Cantoblanco, 28049 Madrid (Spain)
E-mail: felix.zamora@uam.es

Table of Contents

1. Experimental Procedures......3

1.1 Chemicals......3

1.2 Synthesis......3

1.2.1 Synthesis of TFPT monomer......3

1.2.2 Synthesis of IC-COF......3

1.3 Sample Preparation......4

1.3.1 Exfoliating the bulk of IC-COF......4

1.3.2 Anion sensing studies......4

1.4 Characterization......4

1.5 Computational Methods......5

1.5.1 The selection of the preferred interaction sites......5

2. Results and Discussion......8

Figure S1. Calculated images of staggered lattice of IC-COF......8

Figure S2. Calculated images of eclipsed lattice of IC-COF......8

Figure S3. FE-SEM and TEM images of IC-COF......9

Figure S4. FE-SEM images of another product......9

Figure S5. UV-Vis spectra of IC-COF......10

Figure S6. fluorescence spectra of IC-COF......10

Figure S7. Photos of IC-COF suspensions......11

Figure S8. Fluorescence spectra of IC-COF and its constituent monomers......11

Figure S9. Fluorescence spectra of the IC-COF upon treatment with CO_3^{2-} and the linear correlation for the plot of I_0/I 12

Figure S10. The effect of pH......12

Figure S11. The optimized structure of IC-COF......13

Figure S12. The optimized structure of the IC-COF complex......13

Figure S13. The UV-Vis spectra of the IC-COF complex......14

Figure S14. The energy diagram of $[(IC\text{-}COF\text{-}H)^-/HPO_4^{2-}]$. complex......14

Table S1. TGA and DTG values of IC-COF......15

Table S2. Excitation and emission parameters of the IC-COF suspensions......15

Table S3. PI sensing LOD comparison for various probes 15

3. References......16

1. Experimental Procedures

1.1 Chemicals

All chemicals and solvents were of analytical reagent grade and used as received for the synthesis and other experimental sections. All the working solutions were freshly prepared by adequate diluting of the stock solutions to the required concentrations. 1,5-diaminonaphthalene (1,5-DAN), cyanuric chloride (CC), 4-hydroxybenzaldehyde, tetrabutylammonium bromide (TBAB), ethyl acetate, tetrahydrofuran (THF), dimethyl sulfoxide (DMSO), dimethylformamide (DMF), acetone, ethanol, cyclohexane, sodium hydroxide (NaOH), acetic acid (glacial), phosphate ion (Pi), carbonate ion, and other used material were purchased from Merck|Sigma-Aldrich company.

1.2 Synthesis

1.2.1 Synthesis of TFPT monomer

For synthesis of TFPT, initially, a solution of cyanuric chloride (0.92 g, 5 mmol) in acetone (10 ml) was added dropwise to a solution of 4-hydroxybenzaldehyde (2.44 g, 20 mmol) in water (50 ml) with NaOH (0.8g, 20 mmol) and the resulting solution stirred at room temperature for 24 h. The reaction mixture was filtered, and the opaline solid was washed with 10% sodium hydroxide solution (3 × 25 ml) and double distilled water (2 × 25 ml) and then crystallized.[1] Yield, 89%; m.p., 175 °C; elemental analysis calcd (%) for $(C_{24}H_{15}N_3O_6)_n$: C 65.31; H 3.43; N 9.52; found: C 65.04, H 3.75, N 9.44; FT-IR (KBr, cm^{-1}): 3065 (m), 2830 (m), 2740 (m), 1701 (s), 1583 (s), 1494 (s), 1211 (s), 842 (s); ^{1}H NMR (500 MHz, $CDCl_3$, 25°C, TMS): δ=7/26 (d, J = 8/4 Hz, 6H; Ar-H), 7/86 (d, J = 8/4 Hz, 6H; Ar-H), 9/95 (s, 3H; CHO); ^{13}C NMR (125 MHz, $CDCl_3$, 25°C, TMS): δ=191/8 (C=O aldehyde), 172/6 (C=N Triazine), 155 (C aromatic), 134 (C aromatic), 131 (CH aromatic), 122 (CH aromatic).

1.2.2 Synthesis of IC-COF

0.2 mmol (88.3 mg) of the synthesized TFPT was dispersed in 80.0 ml of Milli-Q water and subjected to ultrasonic waves for 15 minutes. 1,5-DAN (0.3 mmol, 47.5 mg) was completely dissolved in 0.5 ml of DMSO and then added to 80.0 ml of pre-heated Milli-Q water. Ensuing, 8.0 ml of glacial acetic acid, as a reaction catalyst, was added to the solution. Both prepared solutions were mixed, and the reaction mixture was exposed to 80 °C for 5 days. The obtained brownish-purple product was filtered and washed

with a large volume of hot ethanol and deionized water, then extracted with Soxhlet under ethanol solvent for 5 days. Finally, the purified precipitate was separated by filtration and dried at ambient temperature. The reaction efficiency was 96%. Elemental analysis calcd (%) for $(C_{34}H_{21}N_5O_3)_n$: C 74.59; H 3.87; N 12.79; found: C 74.99, H 4.01, N 12.33.

1.3 Sample Preparation

1.3.1 Exfoliating the bulk of IC-COF

5 mg of the IC-COF was dispersed in 50 ml of ethanol and exposed to ultrasonic irradiation in a bath sonicator for 2 hr. The solid was then separated from the solvent by centrifuging and dried in an oven at 100 °C for 10 h. The volume of solid increased about 3 times after exposure to ultrasonic irradiation.

1.3.2 Anion sensing studies

In this work, a stock solution of IC-COF was prepared by taking 1 mg of IC-COF in 5 mL of DMSO, sonicated for 30 min to obtain a homogeneous suspension. The sensing experiments were carried out by adding 10.0 μL aqueous solutions of corresponding potassium salts of different anions (Br^-, PO_4^{3-}, NO_3^{1-}, OAc^{2-}, SO_4^{2-}, CO_3^{2-}, ClO_4^-, CrO_4^{2-}, $Cr_2O_7^{2-}$, Cl^-, OH^-, SCN^-, MnO_4^-, F^-, and I^-) and chloride salts of various metals (Mg^{2+}, Ba^{2+}, Al^{3+}, Fe^{2+}, Cu^+, Hg^{2+}, Cd^{2+}, Pb^{2+}, Li^+, and Fe^{3+}) at the concentration of 0.01 mol L^{-1} into 2.0 mL of IC-COF solution, with 1.3 mg L^{-1} concentration, at room temperature and collecting the luminescence data immediately. All the emission spectra of IC-COF suspensions were recorded from 450 to 650 nm upon excitation at 470 nm. The fluorescence quenching efficiency was estimated employing the Stern−Volmer equation, $I_o/I = K_{SV}[M] + 1$, in which I_o and I are the luminescence intensity of IC-COF before and after addition of PO_4^{3-} anion, respectively, K_{SV} represents the quenching constant and [M] is the concentration of PO_4^{3-} anion.

1.4 Characterization

Fourier Transform-Infrared spectra (FT-IR) were acquired on a Jasco-680 plus spectrometer in the scope of 4000-400 cm^{-1} by using KBr pellets. **Powder X-ray diffraction** (PXRD) pattern was measured on a Bruker D8 ADVANCE diffractometer (Germany) using Cu Kα radiation in a 2θ range of 0.2°-30° at room temperature. **Hydrogen-1 nuclear magnetic resonance** (^1H NMR, 500 MHz) and **Carbon-13 nuclear magnetic resonance** (^{13}C NMR,125 MHz) were obtained on a Bruker AVANCE spectrometer in the chloroform-d solvent. The TC-COF nanosheets microstructure was also investigated by **transmission electron microscopy** (TEM) analysis (Philips CM120; Holland, operated at 120 kV with a

point-to-point resolution of 0.1 nm). **X-ray photoelectron spectroscopy** (XPS) analysis was recorded on an X-ray 8025-BesTec XPS system (Germany) with an Al Kα X-ray source (hυ = 1486.6 eV). **Nitrogen adsorption-desorption isotherms** were measured at 77 K using a Japanese BELSORP MINI II surface area and porosity analyzer. **Thermogravimetric analysis** (TGA) was carried out utilizing a Germany STA TA 503 thermogravimetric analyzer at a heating rate of 10 °C/min under N_2 atmosphere. **Ultraviolet–visible** (UV-Vis) spectra were carried out on a Shimadzu UV-2550 spectrophotometer. The fluorescence spectra were recorded on a Shimadzu RF- 5301PC fluorescence spectrometer with the slit width for both excitation and emission was set to 5 nm. Elemental analysis was conducted with a CHN-932 Leco elemental analyzer.

1.5 Computational Methods

To reduce the cost of the calculations, especially in the simulation of the IC-COF complexes absorption spectra, a part of the IC-COF was selected. The selected part structure was optimized employing a corrected dispersion DFT functional (B3LYP-D3)[2] and 6-311++G(d, p) basis set for all atoms (Figure S11a). The LANL2DZ method was used for the Cu^+ atoms. The one-electron density surface of the molecule was calculated at the same level of theory (Figure S11b) to distinguish the positive and negative potential on the molecular surface and propose the best interaction sites for PO_4^{-3} and Cu^+ ions with the IC-COF. The IC-COF-Cu+ and IC-COF-PO43- complexes' initial structure was optimized at the B3LYP-D3/6-311++G(d, p)/LANL2DZ B3LYP-D3/6-311++G(d, p) levels of theory, respectively. The frequency calculations were performed on the optimized structures to select the most stable structure of the IC-COF-Cu^+ complex and confirm their position on the local minimum of their potential energy surfaces. Three hundred excited states were considered in the time-dependent density functional theory (TD-DFT) to calculate the absorption spectra for the most stable structure of the IC-COF-Cu^+ and IC-COF-PO_4^{3-} complexes. All calculations were performed in the DMSO solvent employing the polarized continuum model (PCM). The Gaussian 09 Quantum Chemistry Package was used for all calculations.[3]

1.5.1 The selection of the preferred interaction sites

The calculated map of electrostatic potential (MEP) surface shows that due to the positive electrostatic potential near the unreacted NH_2 groups, these groups are the best sites for interaction with the PO_4^{3-} via the hydrogen bonds (Figure S12a). The phenyl rings, O, and N atoms are the appropriate positions for the interaction of Cu^+ with IC-COF in the selected part structure. The optimized structures of these complexes have shown in Figures S12b to S12f. The relative standard Gibbs free energies of the IC-COF-Cu^+ complexes have shown in the mentioned figures. Based on these values, structures *b* and *f* are favorable for the IC-COF-Cu^+ complex. In these structures, the Cu^+ ion is stabilized *via* the simultaneous interaction

with the lone pair electrons of heteroatoms and π electrons of the phenyl ring. Structure *b*, as the most stable structure of the IC-COF-Cu^+ complex, structure *a* was selected to calculate the absorption spectra.

The bare IC-COF absorption spectrum observed two very close absorption lines at 439.7 and 441.2 nm. The main electronic configurations for both lines are the same and related to the one-electron orbital excitations, including HOMO→LUMO+1, HOMO→LUMO+2, HOMO-1→LUMO+1, and HOMO-1→LUMO+2. The molecular orbitals of the bare IC-COF have shown in Figure 8a. As can be seen, the HOMO and HOMO-1 of the molecule are mainly related to the diaminonaphthalene part. The LUMO orbital of the IC-COF has been localized on the benzaldehyde section. The electron density of its LUMO+1 and LUMO+2 orbitals has been mainly distributed on both phenyl rings, the linkers between the diaminonaphthalene and s-triazine ring. Therefore, both the bare IC-COF absorption lines are related to the electron excitation from the naphthalene part to the phenyl ring in the TFPT structure. Due to the slight energy difference between the HOMO and HOMO-1, they can be considered degenerate orbitals. Similarly, the LUMO+1 and LUMO+2 are also degenerate.

The interaction of Cu^+ with the IC-COF did not change the IC-COF-Cu^+ absorption spectrum compared to the bare IC-COF, and only the interval between the two absorption lines (located at 450.1 and 445.9 nm) increased slightly. The main electronic configuration of the first (450.1 nm) and second (445.9 nm) absorption lines of IC-COF-Cu^+ complex are mostly related to the HOMO-1→LUMO+1 and HOMO→LUMO+2 excitations, respectively. It seems that the presence of the Cu^+ decreases the number of one-electron orbital excitations in the main electronic configurations of both absorption lines compared to the bare IC-COF. Comparing the LUMO+1 and LUMO+2 in the IC-COF-Cu^+ complex with the bare IC-COF shows the effect of Cu^+ on the IC-COF electronic structure (Figures 8a and 8b). In the presence of Cu^+, the electron density of these orbitals is mainly localized on the TFPT phenyl ring, which is the linkage between the diaminonaphthalene and s-triazine ring. Since the Cu^+ interacts with the heteroatoms and phenyl ring of the TFPT, the considerable energy change (~-0.6 eV) occurs for the LUMO orbital of the IC-COF-Cu^+ complex compared to the bare IC-COF; nevertheless, this interaction has not disturbed the degeneracy between the orbitals.

The interaction of the PO_4^{3-} with the IC-COF significantly reduces the intensity of the located broad peak at 400-600 nm due to the shift of one of the two IC-COF absorption lines to the higher wavelength (Figure 7a). The main electronic configuration of the IC-COF-PO_4^{3-} absorption lines at 439.7 and 522.0 nm are related to HOMO-4→LUMO+1 and HOMO-3 → LUMO+2, respectively. Figure 8c shows the energy diagram of the IC-COF-PO_4^{3-} molecular orbitals related to these absorption lines. The comparison between Figures 8b and 8c shows that the HOMO-4 and HOMO-3 orbitals of the IC-COF-PO_4^{3-} complex

correspond to the HOMO-1 and HOMO of the IC-COF-Cu^+ complexes, respectively. Contrary to what was seen in the IC-COF-Cu^+ complexes orbital diagram, the HOMO-4 and HOMO-3 orbitals of the IC-COF-PO_4^{3-} complex are non-degenerate. The same comparison between the IC-COF-PO_4^{3-} and IC-COF shows that the HOMO-4 and HOMO-3 of the IC-COF-PO_4^{3-} complex correspond to the HOMO-1 and HOMO of the IC-COF, respectively. The PO_4^{3-} contribution in the HOMO-3 orbital of the IC-COF-PO_4^{3-} complex could be due to the interaction of PO_4^{3-} with the NH_2 group, which leads to a reduction in the degeneracy between the HOMO-3 and HOMO-4 orbitals. Similarly, this interaction removes the degeneracy of the LUMO and LUMO+1 orbitals in the IC-COF-PO_4^{3-} complex. The HOMO-2, HOMO-1, and HOMO of the IC-COF-PO_4^{3-} complex are mainly related to the PO_4^{3-} because of the electron density localization on this fragment. The LUMO orbital energy in the IC-COF-PO_4^{3-} complex did not change much compared to the bare IC-COF (Figure 8a) because PO_4^{3-} interacts with the NH_2 group. In contrast, the LUMO orbital is related to the aldehyde part of the structure.

The possibility of proton transfer from the NH_2 group of the IC-COF to the PO_4^{3-} can also take place. Comparison of the [(IC-COF-H)$^-$/HPO_4^{2-}] calculated spectrum with IC-COF shows that the proton transfer shifts the maximum of the located peak in the range of 400 to 500 nm to the lower wavelength (Figure 7). The molecular orbital diagram of the [(IC-COF-H)$^-$/HPO_4^{2-}] complex has been shown in Figure S14. Comparison Figure S14 with the molecular orbital diagram of the IC-COF-PO_4^{3-} complex (Figure 8c) shows that the proton transfer change the energy order of the occupied molecular orbitals. The HOMO-1 and HOMO-2 of the [(IC-COF-H)$^-$/HPO_4^{2-}] complex are degenerate. The HOMO of [(IC-COF-H)$^-$/HPO_4^{2-}] complex has been localized on the diaminonaphthalene ring while the HOMO of the IC-COF-PO_4^{3-} complex has been localized on PO_4^{3-}. The LUMO of two complexes are the same.

2. Results and Discussion

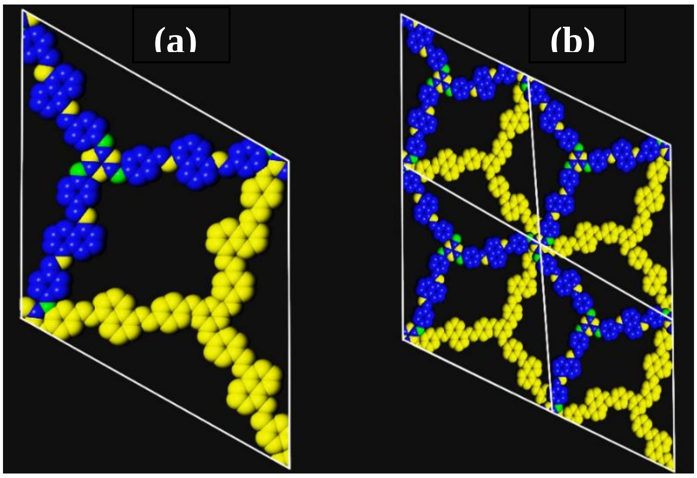

Figure S1. (a) Unit cell and (b) staggered crystal lattice packing of IC-COF.

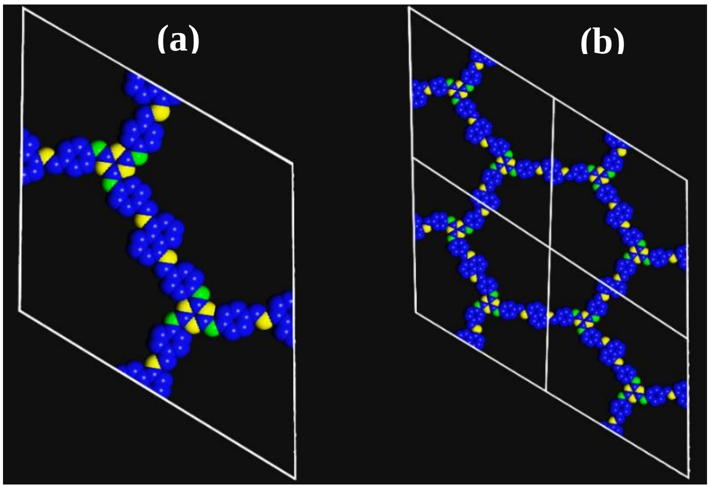

Figure S2. (a) Unit cell and (b) eclipsed crystal lattice packing of IC-COF.

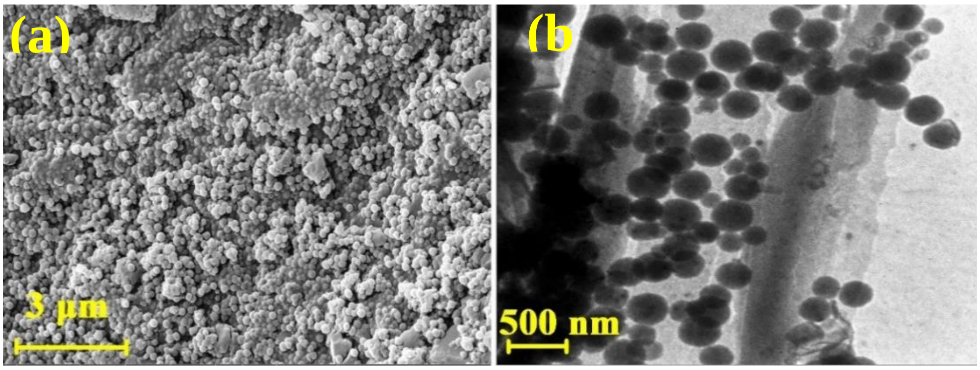

Figure S3. (a) FE-SEM and (b) TEM images of IC-COF.

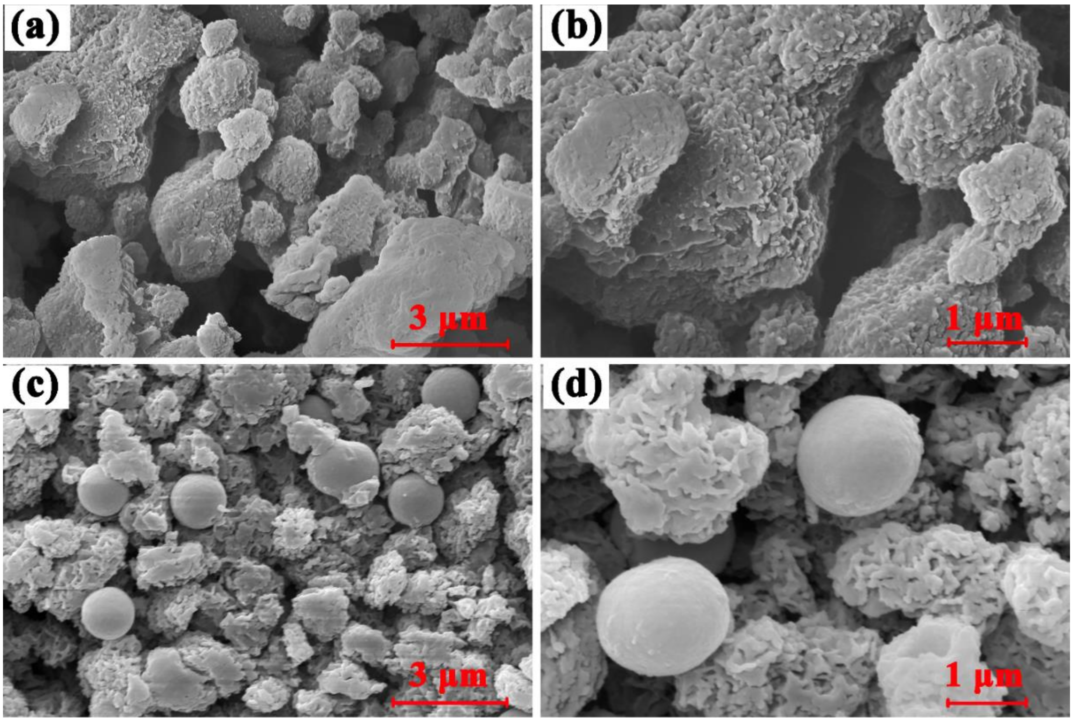

Figure S4. (a, b) FE-SEM images of the obtained product using the reflux system in mesitylene and 1,4-dioxane solvents (1: 1) in the presence of glycolic acetic acid; (c, d) FE-SEM images of the solvothermal synthesis method product by using of the mixture of mesitylene and 1,4-dioxane solvents in equal volume ratios and diluted acetic acid as a catalyst.

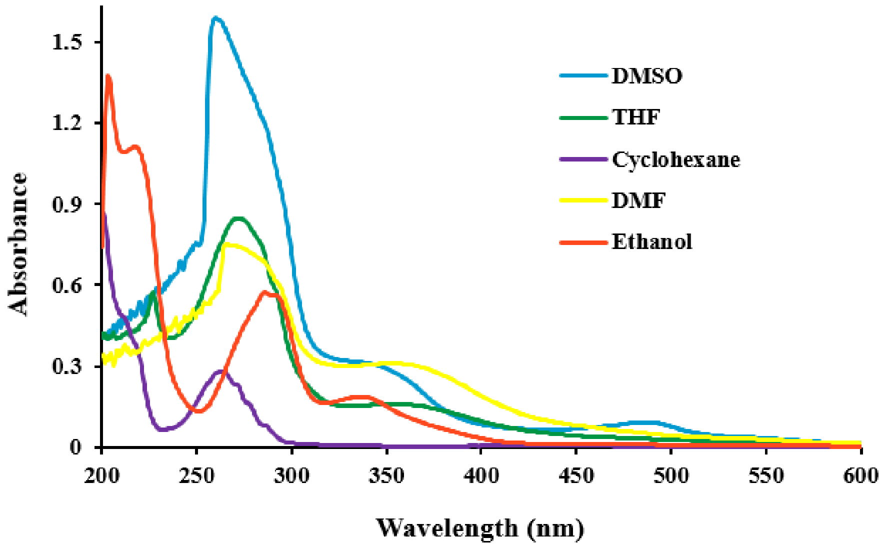

Figure S5. The UV-Vis spectra of IC-COF in various solvents. (For interpretation of the references to color in this figure legend, the reader is referred to the web version of this article).

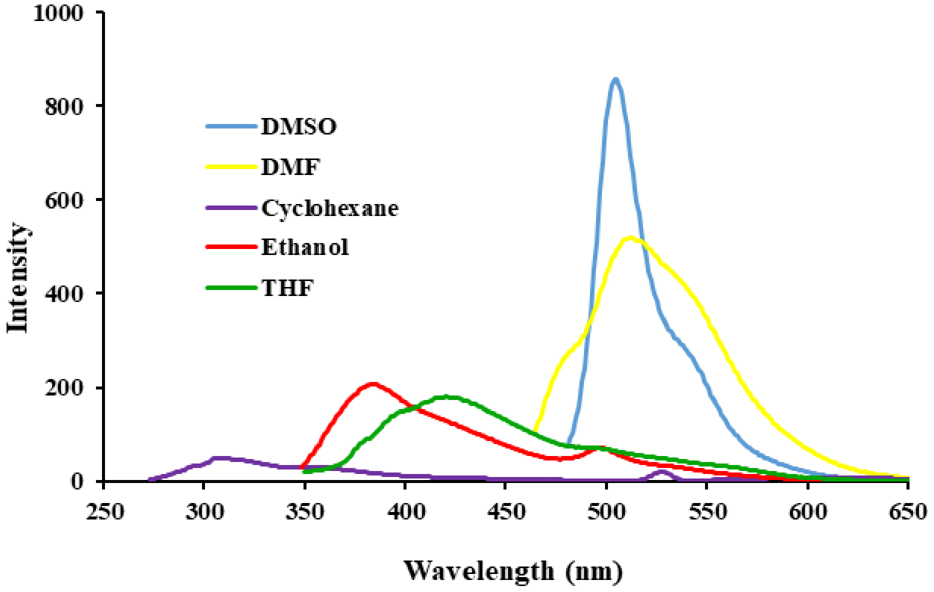

Figure S6. IC-COF fluorescence spectra at the optimal excitation wavelength of each solvent (For interpretation of the references to color in this figure legend, the reader is referred to the web version of this article).

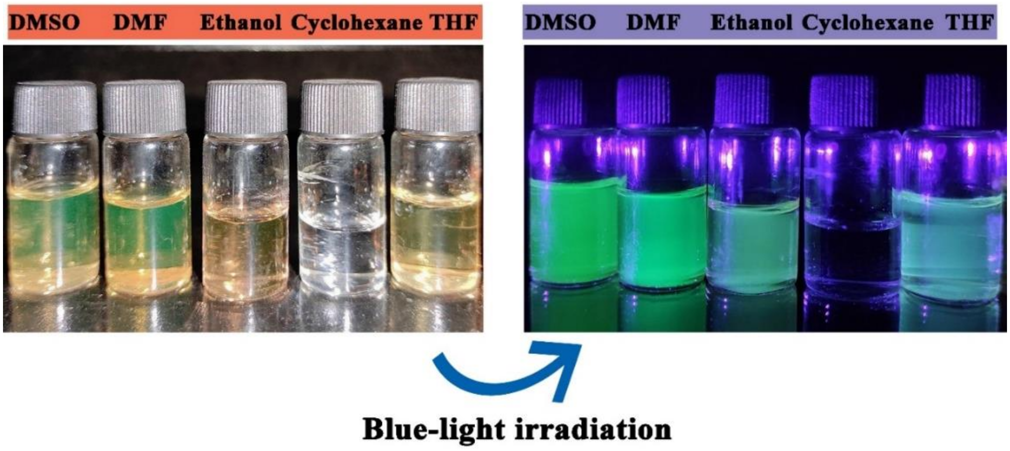

Figure S7. Photos of IC-COF suspension in various solvents, before and after irradiation with 470 nm light

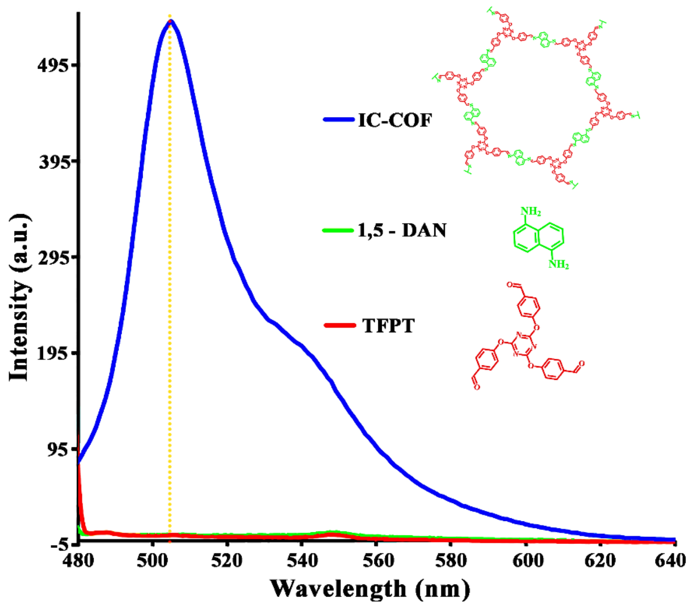

Figure S8. Fluorescence spectra of IC-COF and its constituent monomers in the dispersion solution of DMSO.

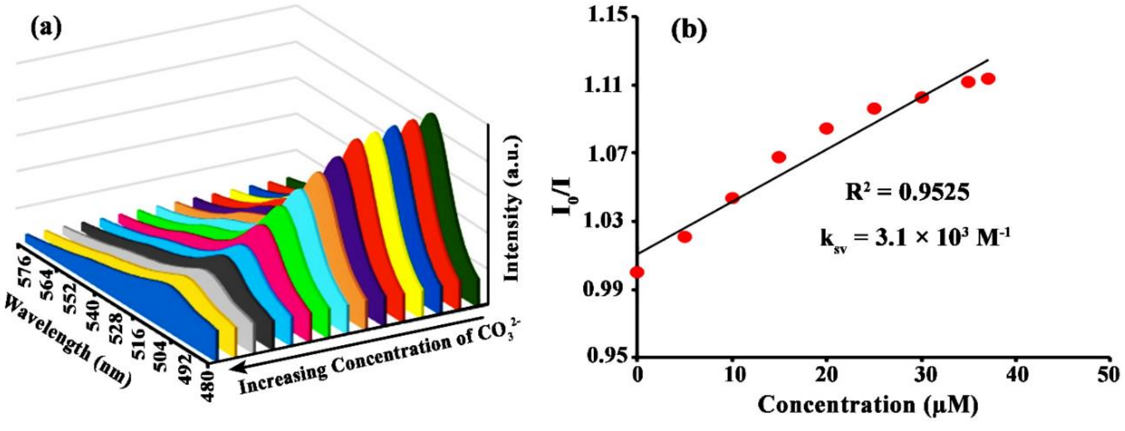

Figure S9. (a) Fluorescence spectra of the IC-COF suspensions upon the gradual treatment with CO_3^{2-} (0–37 μM), excited at 470 nm; (b) the linear correlation for the plot of I_0/I as a function of CO_3^{2-} concentration

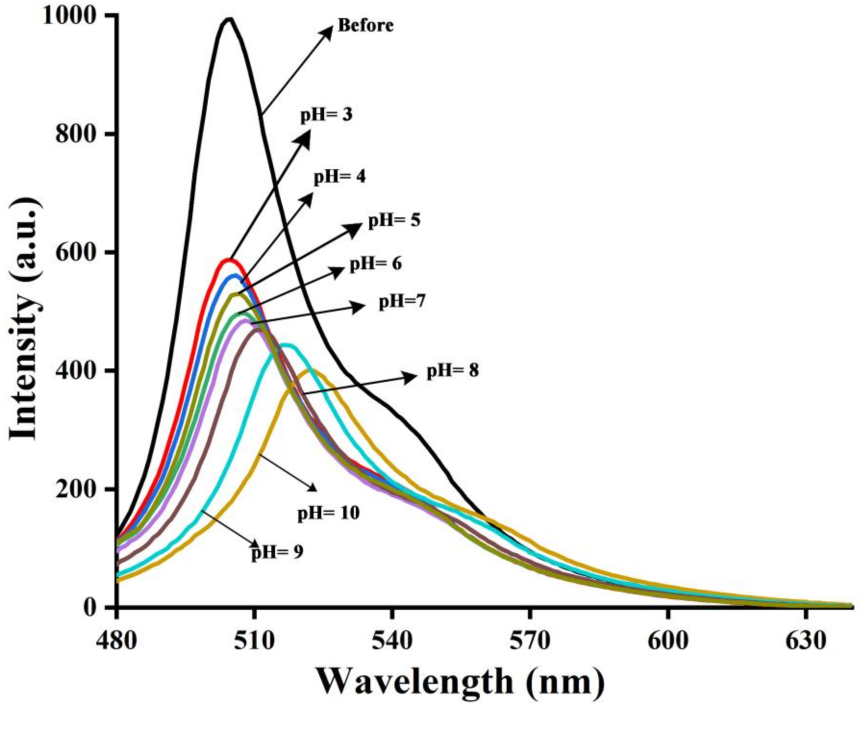

Figure S10. The effect of pH on the quenching of IC-COF fluorescence emission by phosphate ion in the presence of various pHs.

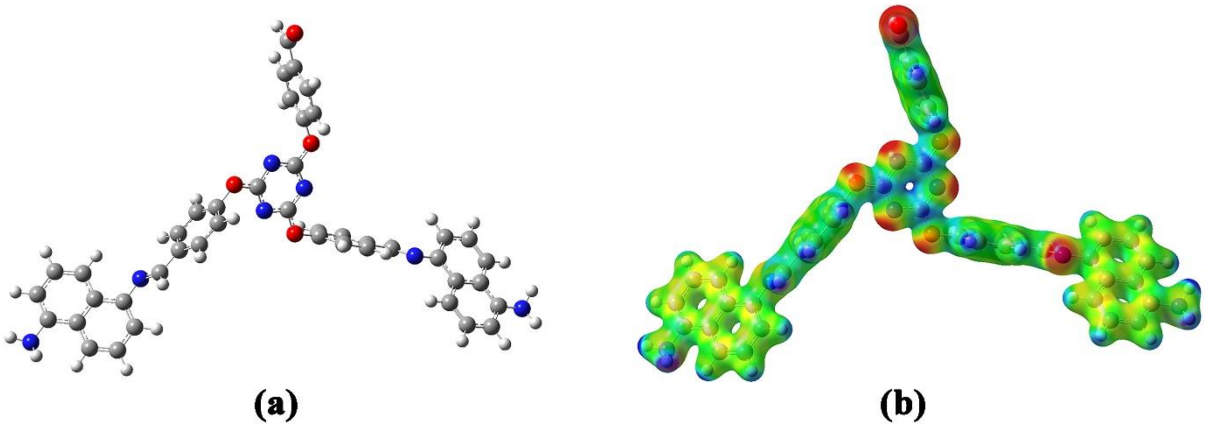

Figure S11. (a) The optimized structure of the selected part of the IC-COF (b) the calculated one-electron density surface (MEP) of the optimized selected part of the IC-COF. The blue parts show the regions with positive potentials. The electron density increases when the color changes from blue to red.

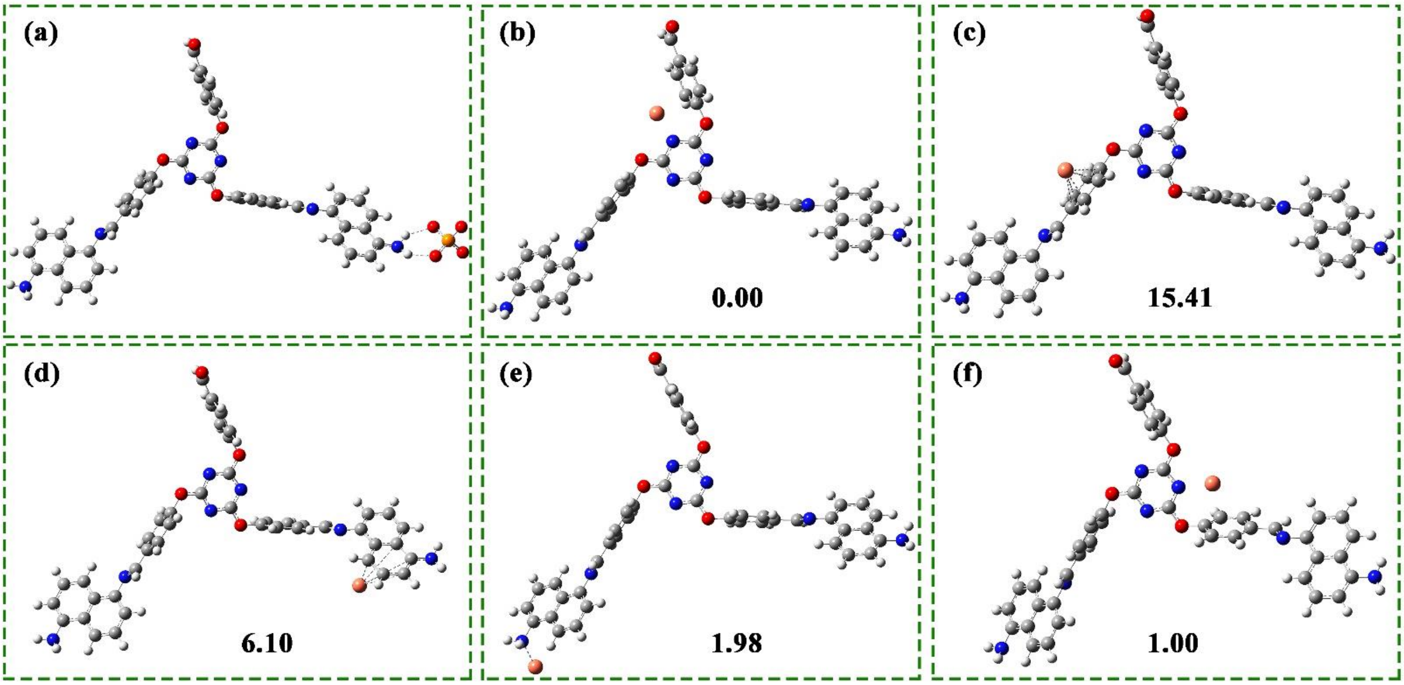

Figure S12. (a) The optimized structure of the complex of the selected IC-COF with PO_4^{-3} (IC-COF-$PO4^{-3}$). (b), (c), (d), (e), and (f) are the optimized structures of the different complexes of the selected IC-COF with Cu^+ (IC-COF-Cu^+). The numbers show the relative Gibbs free energy of the IC-COF-Cu^+ complexes in kcal/mol.

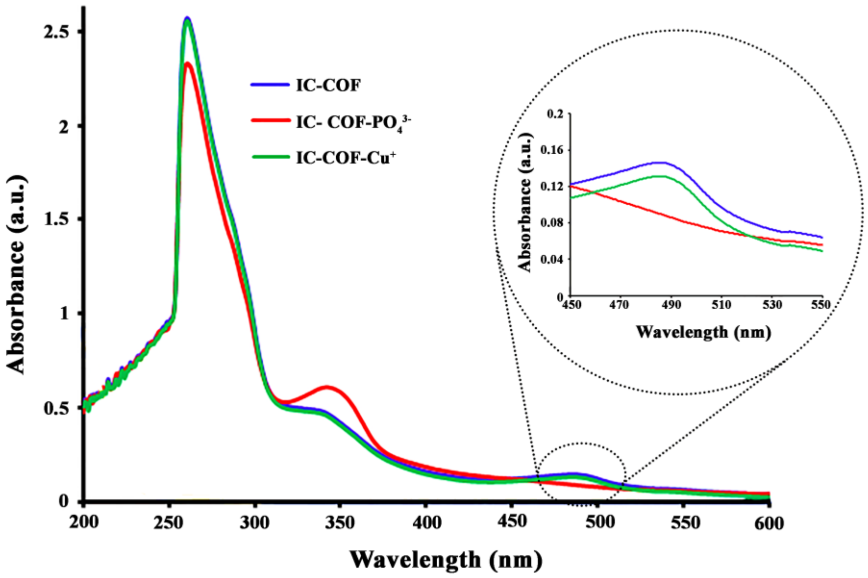

Figure S13. The UV-Vis spectra of IC-COF composition in the presence and absence of PO_4^{3-} and Cu^+. (For interpretation of the references to color in this figure legend, the reader is referred to the web version of this article).

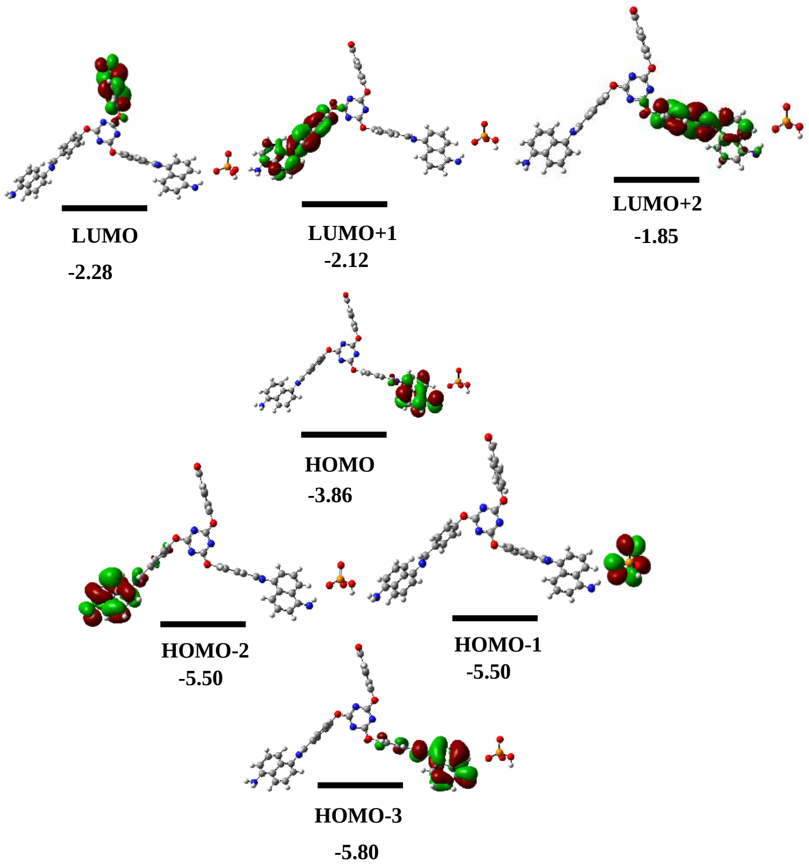

Figure S14. The molecular orbitals energy diagram of $[(\text{IC-COF-H})^-/HPO_4^{2-}]$. The numbers are the orbital energies in eV.

Table S1. TGA and DTG specific values of IC-COF under argon atmosphere.

Sample	T5 wt% (℃)	Tmax (℃)	Residue (wt%)
IC-COF	215	440	52

Table S2. Excitation and emission parameters of the IC-COF in different solvents.

Solvents	DMSO	DMF	Ethanol	THF	Cyclohexane
Optimal excitation wavelength (nm)	470	450	336	340	283
IC-COF concentration (ppm)	1.3	10	10	10	10
Emission wavelength (nm)	505	511	385	421	307

Table S3. Comparison of LOD of various probes for sensing phosphate ions.

Entries	Probe	LOD (μM)	Reference
1	Mn: Zn Te/ZnSe QDs	0.20	[4]
2	GQD-Eu3+	0.10	[5]
3	NMOF	0.06	[6]
4	Methyl 2-[(4-methyl-2-oxo-2H-chromen-7-yl)oxy]propanoate	0.81	[7]
5	C-dots	0.80	[8]
6	BDC-NH2	1.25	[9]
7	IC-COF	0.61	This work

3. References

(1) Zhao, Y.; King, G.; Kwan, M. H. T.; Blacker, A. J. A Mild and Selective Method for the Catalytic Hydrodeoxygenation of Cyanurate Activated Phenols in Multiphasic Continuous Flow. *Org. Process Res. Dev.* **2016**, *20* (11), 2012–2018. https://doi.org/10.1021/acs.oprd.6b00314.

(2) Torres, E.; Dilabio, G. A. A (Nearly) Universally Applicable Method for Modeling Noncovalent Interactions Using B3LYP. *J. Phys. Chem. Lett.* **2012**, *3* (13), 1738–1744. https://doi.org/10.1021/jz300554y.

(3) Frisch, M.; Clemente, F. Gaussian 09, Revision A. 01, MJ Frisch, GW Trucks, HB Schlegel, GE Scuseria, MA Robb, JR Cheeseman, G. *Scalmani, V. Barone, B. Mennucci, GA Petersson, H. Nakatsuji, M. Caricato, X. Li, HP Hratchian, AF Izmaylov, J. Bloino, G. Zhe* **2009**.

(4) Song, Y.; Li, Y.; Liu, Y.; Su, X.; Ma, Q. Highly Sensitive and Selective Detection of Phosphate Using Novel Highly Photoluminescent Water-Soluble Mn-Doped ZnTe/ZnSe Quantum Dots. *Talanta* **2015**, *144*, 680–685. https://doi.org/10.1016/j.talanta.2015.07.025.

(5) Bai, J. M.; Zhang, L.; Liang, R. P.; Qiu, J. D. Graphene Quantum Dots Combined with Europium Ions as Photoluminescent Probes for Phosphate Sensing. *Chem. - A Eur. J.* **2013**, *19* (12), 3822–3826. https://doi.org/10.1002/chem.201204295.

(6) Ma, Y.; Zhang, Y.; Li, X.; Yang, P.; Yue, J.-Y.; Jiang, Y.; Tang, B. Linker-Eliminated Nano Metal–Organic Framework Fluorescent Probe for Highly Selective and Sensitive Phosphate Ratiometric Detection in Water and Body Fluids. *Anal. Chem.* **2020**, *92* (5), 3722–3727. https://doi.org/10.1021/acs.analchem.9b04958.

(7) Guo, L. E.; Zhang, J. F.; Liu, X. Y.; Zhang, L. M.; Zhang, H. L.; Chen, J. H.; Xie, X. G.; Zhou, Y.; Luo, K.; Yoon, J. Phosphate Ion Targeted Colorimetric and Fluorescent Probe and Its Use to Monitor Endogeneous Phosphate Ion in a Hemichannel-Closed Cell. *Anal. Chem.* **2015**, *87* (2), 1196–1201. https://doi.org/10.1021/ac503818p.

(8) Xu, J.; Zhou, Y.; Cheng, G.; Dong, M.; Liu, S.; Huang, C. Carbon Dots as a Luminescence Sensor for Ultrasensitive Detection of Phosphate and Their Bioimaging Properties. *Luminescence* **2015**, *30* (4), 411–415. https://doi.org/10.1002/bio.2752.

(9) Yang, J.; Dai, Y.; Zhu, X.; Wang, Z.; Li, Y.; Zhuang, Q.; Shi, J.; Gu, J. Metal–Organic Frameworks with Inherent Recognition Sites for Selective Phosphate Sensing through Their Coordination-Induced Fluorescence Enhancement Effect. *J. Mater. Chem. A* **2015**, *3* (14), 7445–7452. https://doi.org/10.1039/C5TA00077G.
